# Status of macromolecular crystallography beamlines at SSRF

**DOI:** 10.1107/S1600577524011597

**Published:** 2025-01-01

**Authors:** Huating Kong, Huan Zhou, Qin Xu, Ke Liu, Kunhao Zhang, Xingya Wang, Weiwei Wang, Zhijun Wang, Sisheng Wang, Yuzhu Wang, Lin Tang, Feng Yu, Jianhua He, Qisheng Wang

**Affiliations:** a Shanghai Synchrotron Radiation Facility, Shanghai Advanced Research Institute Chinese Academy of Sciences Shanghai201204 People’s Republic of China; b Shanghai Institute of Applied Physics Chinese Academy of Sciences Shanghai201800 People’s Republic of China; c The Institute for Advanced Studies Wuhan University Wuhan430072 People’s Republic of China; University of Manchester, United Kingdom

**Keywords:** Shanghai Synchrotron Radiation Facility, SSRF, macromolecular crystallography beamlines

## Abstract

Shanghai Synchrotron Radiation Facility (SSRF) is a third-generation 3.5 GeV synchrotron facility located on the Chinese mainland, operational for user applications since 2009. With the completion of its Phase II project this year, SSRF now supports over 40 experimental stations across various research fields. For the structural biology community, there are three macromolecular crystallography (MX) beamlines (BL02U1, BL17UM and BL10U2) and one endstation at the white X-ray beamline (BL03HB) managed by SSRF to meet the needs of both academic and industrial users seeking to determine macromolecular crystal structures. The MX group at SSRF is dedicated to continuously upgrading these beamlines in terms of technology and scientific capabilities. This paper reports on the current status of all the MX beamlines at SSRF and discusses emerging trends.

## Abstract 2

The current status and emerging trends of the MX beamlines at SSRF are described.

## 1. Introduction

Since the establishment of the first macromolecular X-ray crystallography (MX) beamline (Phillips *et al.*, 1976 ▸) at the Stanford Synchrotron Radiation Laboratory (SSRL), the number of beamlines dedicated to the crystallographic determination of biological macromolecules has expanded significantly worldwide. According to statistics from the Biosync website (https://biosync.rcsb.org/), there are over 130 MX beamlines currently in operation. These facilities serve as crucial platforms for structural determination and functional research, with more than 80% of structures in the Protein Data Bank (PDB, https://www.rcsb.org) resolved using MX techniques.

Shanghai Synchrotron Radiation Facility (SSRF) is the first third-generation synchrotron facility on the Chinese mainland, officially commencing operations in 2009 (Cyranoski, 2009 ▸). The initial Phase I project included only seven beamlines, one of which is the MX beamline BL17U1. The operational launch of this beamline attracted numerous research groups to conduct structural biology studies in China. The substantial demand for beam time has prompted the construction of five beamlines with six endstations at the National Facility for Protein Science (Shanghai) (Zhang *et al.*, 2019 ▸; Jiang *et al.*, 2009 ▸), as well as the subsequent establishment of three new MX beamlines in the Phase II project. Currently, there are three monochromatic MX beamlines and one white-beam Laue MX beamline in operation.

The SSRF beamlines typically operate for approximately 5500 h each year, with over 80% of beam time allocated to public users. Since 2009, the user community has consistently expanded and currently remains stable. There are options for regular, fast-track and long-term proposals available for beam-time applications. The recent activation of new beamlines has significantly increased the availability of beam time. Additionally, the development of cryo-electron microscopy (cryo-EM) has attracted a large number of users from the MX community. Beamlines for drug discovery based on structure and serial crystallography using synchrotron radiation (SSX) are currently under development.

This review provides a comprehensive introduction to the MX beamlines at SSRF. The first MX beamline, designated BL17U1, served users from 2009 to 2021 before being relocated to BL02U1 to accommodate the establishment of BL17UM, which is optimized for membrane protein research. The beamline BL10U2, designed for viral-related studies, offers biosafety level-2 protection. Additionally, a white-beam MX endstation on the beamline BL03HB has been established for dynamic structural studies. The characteristics of these beamlines and the contributions of the user community will be discussed in detail.

## 2. The MX beamlines

**Figure 1:**
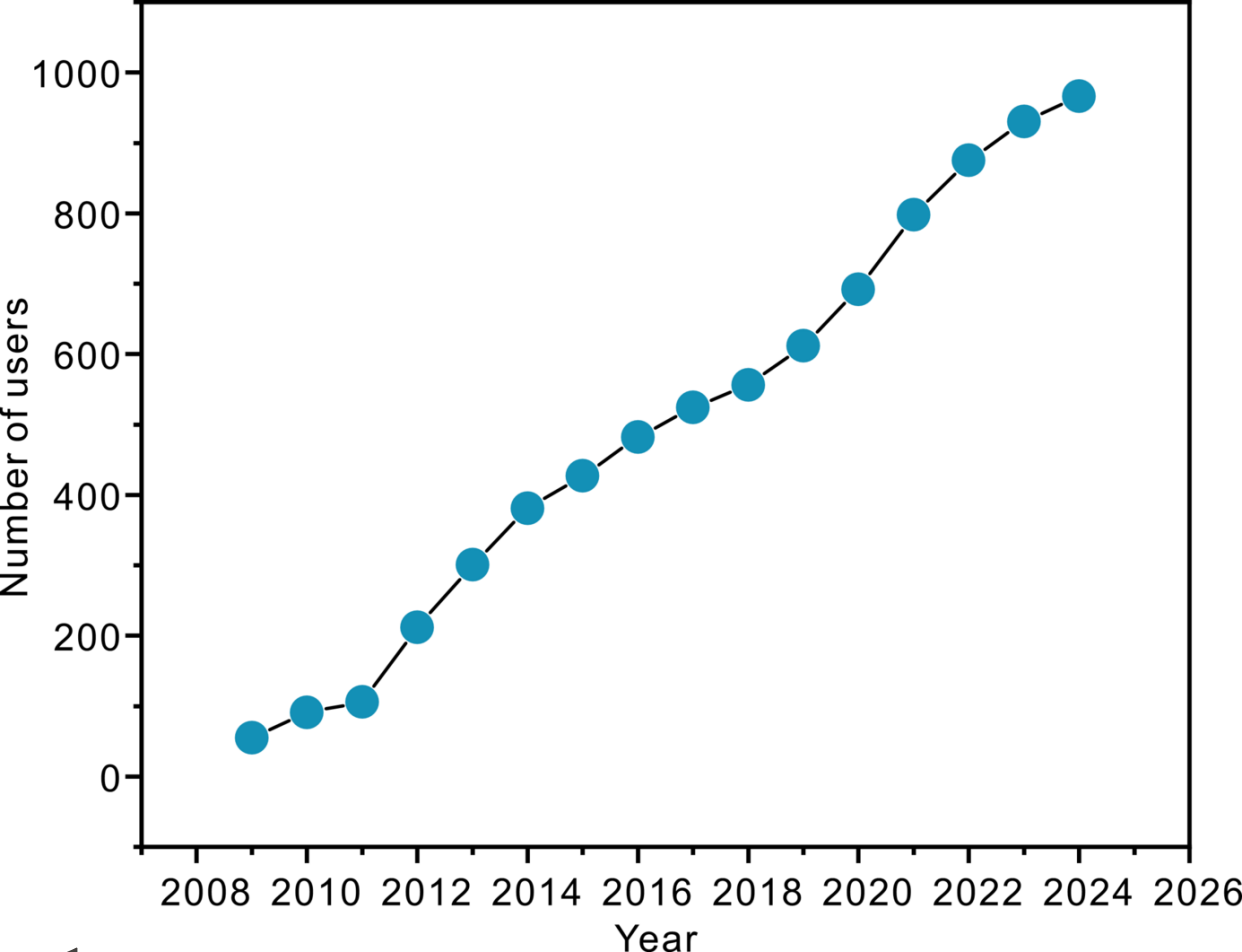
The number of users who have utilized the MX beamlines at SSRF.

Since the opening of the first MX beamline, BL17U1, at SSRF, the user community has experienced rapid growth, increasing from 40 research groups to nearly 300. The number of users who have utilized the MX beamlines has also been continually and significantly increasing (Fig. 1 ▸). This surge in demand for beam time has prompted proposals for additional MX beamlines. Currently, there are four MX beamlines in operation at SSRF, as illustrated in Table 1 ▸.

### 2.1. BL17U1, the first MX beamline

BL17U1 was the first MX beamline at SSRF, designed to serve the research community with general-purpose capabilities (Wang *et al.*, 2015 ▸). The beamline was designed to have a high-flux beam with small beam divergence and reasonable beam size (Nave, 1999 ▸; Helliwell, 1992 ▸). By leveraging the strengths of third-generation synchrotron light sources, BL17U1 provided essential infrastructure for high-resolution structural studies of biological macromolecules. The design prioritized brightness by minimizing the number of components in the light source while focusing the beam to tens of micrometres. BL17U1 utilized an in-vacuum undulator (IVU) with 80 periods of 25 mm-long magnets as its light source. The optical configuration included a double-crystal monochromator (DCM), which narrowed the energy bandwidth of the X-ray beam, and a horizontal-reflecting toroidal mirror focusing the beam in both horizontal and vertical directions. This efficient design enabled BL17U1 to deliver a high photon flux exceeding 10^12^ photons s^−1^ and a focused full width at half-maximum (FWHM) of 67 µm × 23 µm, with a beam divergence characterized by FWHM (H × V) values of 0.3 mrad × 0.1 mrad, within the 5–18 keV energy range.

**Figure 2:**
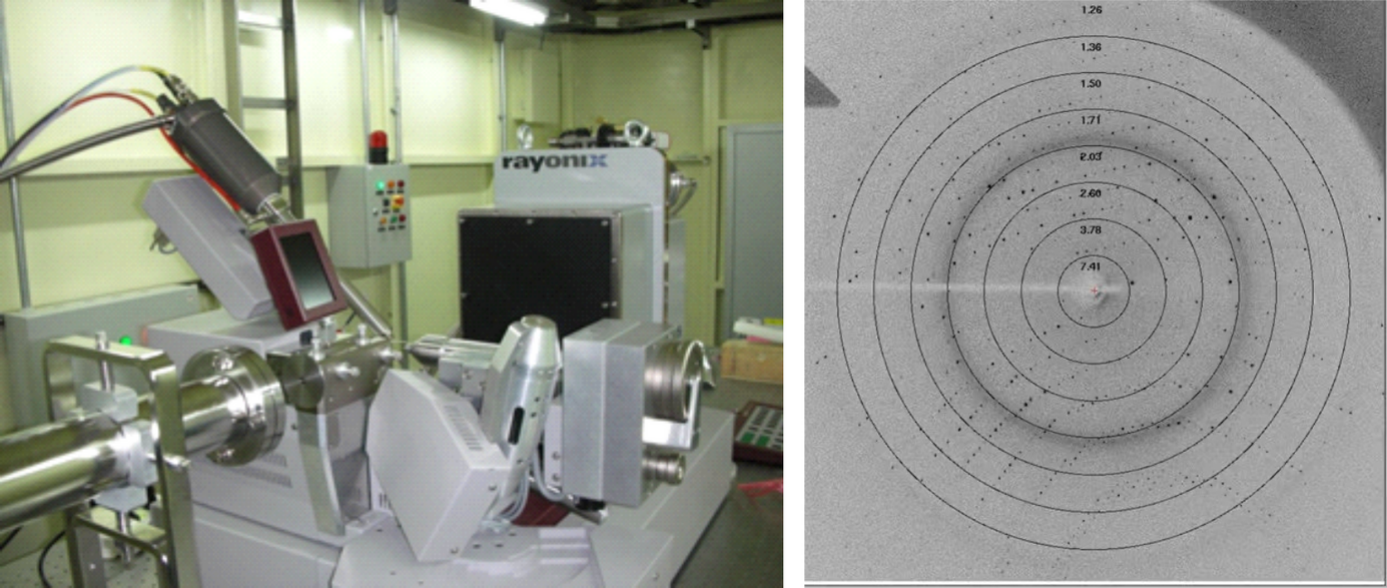
The setup of the endstation at 2009 (left) and the first X-ray diffraction image of lysozyme crystals (right) obtained from BL17U1.

**Figure 3:**
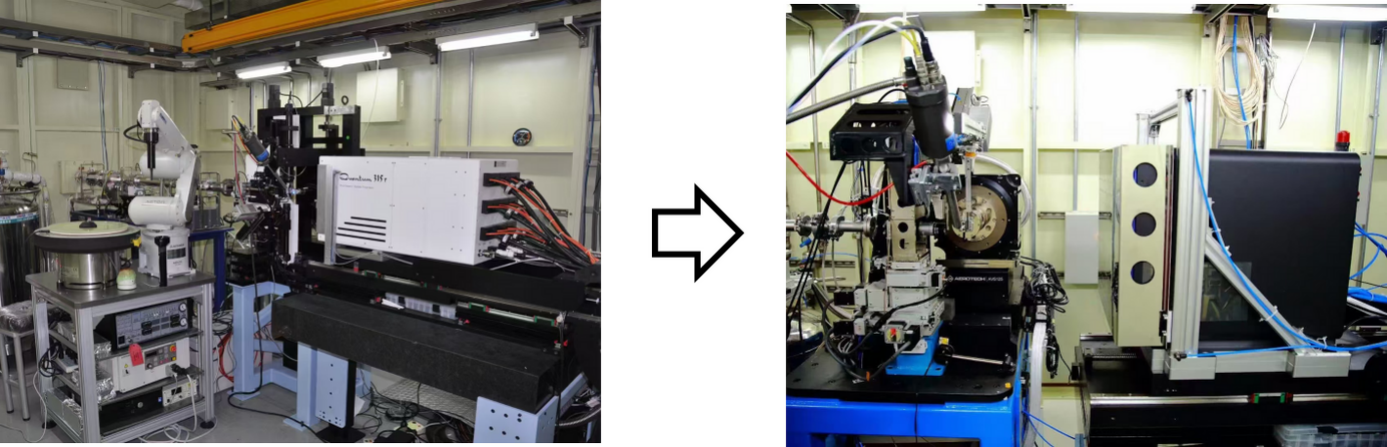
Upgrade of the critical equipment in the BL17U1 endstation.

The endstation of BL17U1 had undergone continuous upgrades and improvements since its opening in 2009. Initially constrained by budget limitations and a lack of experience, it utilized the MarDTB diffractometer and a Mar 225 CCD detector. The first dataset collected was from lysozyme (Fig. 2 ▸). One year after its opening, enhancements were made, including the acquisition of a diffractometer from Crystal Logic Inc., a sample changer from Rigaku and a large-area Q315r CCD detector from ADSC to improve experimental efficiency. With assistance from SSRL, the *Bluice* software was integrated to provide an automated system for experimental operations (Wang *et al.*, 2012 ▸). After nearly a decade of high-intensity usage, the risk of shutdown for the ADSC detector and goniometer significantly increased. This prompted an upgrade (Wang *et al.*, 2018 ▸) (Fig. 3 ▸), which involved a custom-built goniometer and an Eiger X 16M pixel detector, along with the successful replacement of *Bluice* with a new home-developed software called *Finback* (Yu *et al.*, 2024 ▸). Additionally, to facilitate high-throughput data analysis, a data processing pipeline known as Aqua­rium (Yu *et al.*, 2019 ▸) was developed during this period.

To support the construction of a new high-performance membrane protein beamline, BL17U1 had been relocated to BL02U1, which shares the straight section with the BL02U2 beamline due to limited insertion ports. BL17U1 was shut down in February 2021, accumulating a total of approximately 45000 h of user-utilized time. Through the support of this beamline, 4474 structures have been resolved and deposited in the PDB as of 2020.

### 2.2. BL02U1, a relocated beamline of BL17U1

**Figure 4:**
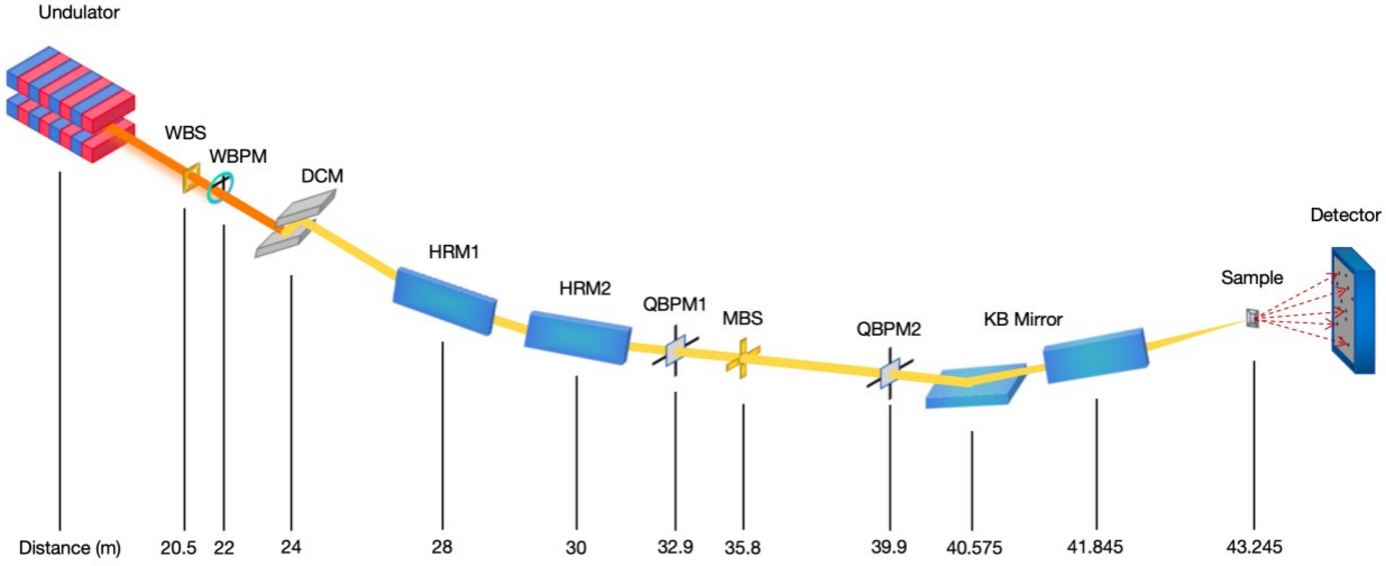
Schematic layout of the BL02U1 beamline. WBS: white beam slit; WBPM: wire beam position monitor; HRM: horizontal reflecting mirrors; QBPM: quadrant beam position monitors; MBS: monochromatic beam slit.

To enhance the experimental capacity for handling micrometre-sized crystals and to increase the utilization rate of the straight-section resources of the SSRF storage ring, the BL17U1 beamline was redesigned for micro-focusing as part of the SSRF Phase II beamline project. It was decommissioned on 8 February 2021, and subsequently rebuilt as BL02U1. The BL02U1 beamline (Liu *et al.*, 2023 ▸) employs a vertical-reflecting DCM to monochromatize the beam generated by an IVU comprising 69 periods of 22 mm-long magnets. The optical design features a single-stage demagnification geometry utilizing a pair of Kirkpatrick–Baez (K-B) mirrors to focus the beam onto a micrometre-sized spot on the sample. Additionally, two flat mirrors positioned downstream of the DCM reflect the beam by 7 mrad in the horizontal direction before it reaches the K-B focusing mirrors, ensuring adequate separation from the neighboring canted beamline, BL02U2 (Gu *et al.*, 2024 ▸) (Fig. 4 ▸). The BL02U1 beamline is capable of delivering a spot size of 11.6 × 4.8 µm within the 6–16 keV energy range, with a photon flux exceeding 10^12^ photons s^−1^ (Table 2 ▸).

**Figure 5:**
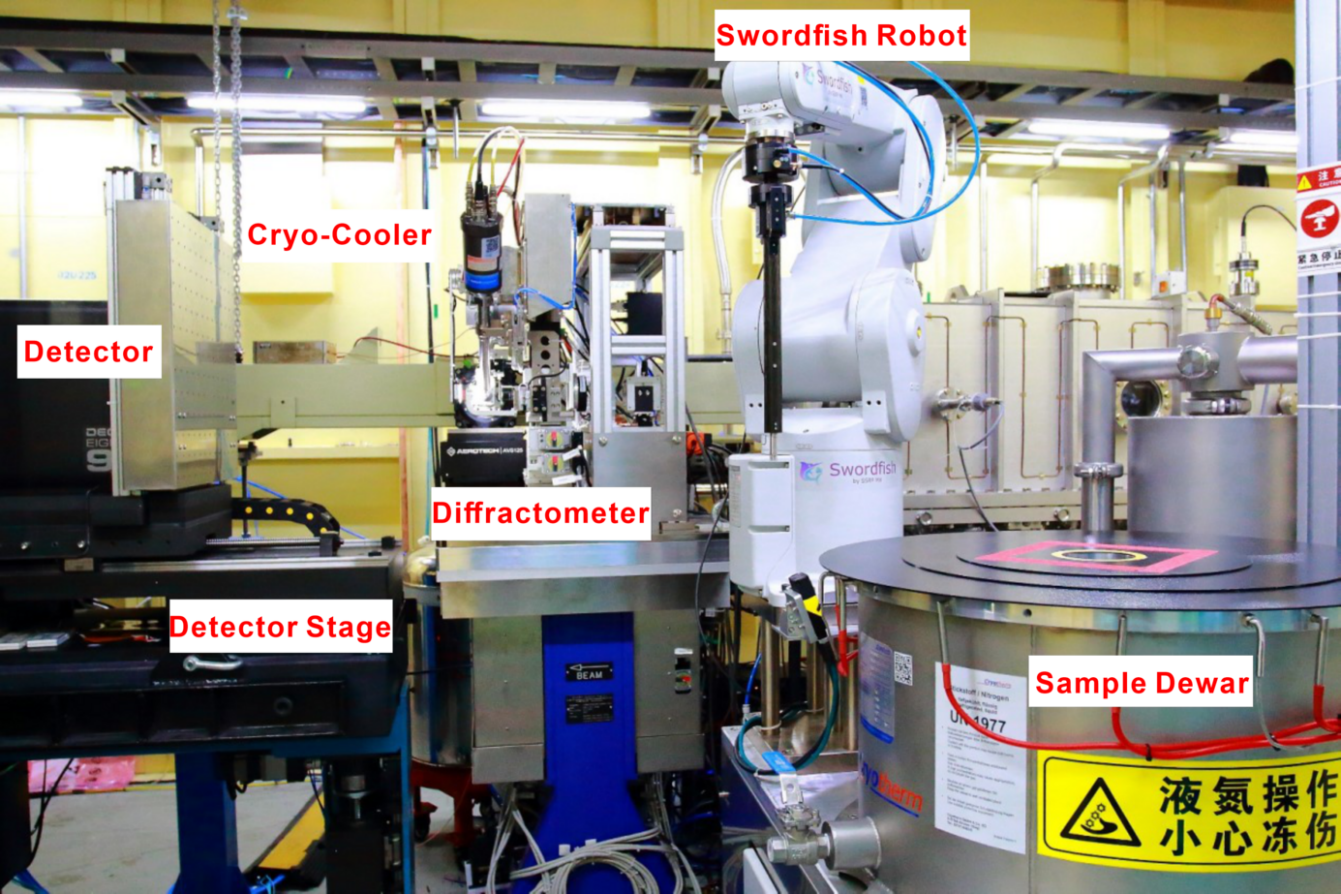
Endstation of the BL02U1 beamline.

The experimental station of BL02U1 is equipped with a single-horizontal-axis diffractometer and an Eiger2 S 9M detector (Dectris Ltd), enabling various MX experiments such as multi-wavelength anomalous diffraction (MAD), single-wavelength anomalous diffraction (SAD), molecular replacement (MR) and isomorphous replacement (IR). To facilitate sample automation, the station features a six-axis robotic arm and a Dewar with 21 unipuck docking positions. To ensure the stable operation of the experimental equipment, the hutch is maintained at a constant temperature of 24°C and a humidity level of approximately 30%. During experiments, samples are preserved in a dry environment at 100 K (Fig. 5 ▸).

Since its opening to users in June 2021, BL02U1 has provided a total of 9580 h of beam time as of August 2024, supporting over 1000 user experiments conducted by 269 research groups from 148 institutions. Through the support of this beamline, 396 structures – including proteins, DNA and RNA – have been resolved and deposited in the PDB to date.

### 2.3. BL10U2, a MX beamline with biosafety level 2

Crystals obtained from live, non-inactivated viruses retain the physiological activity and binding specificity of surface proteins, thereby providing a more accurate representation of the virus’s true structure in its active state. Consequently, the direct resolution of crystal structures from live viruses is crucial for the development of antiviral antibodies and drugs, necessitating support from MX beamlines equipped with biosafety features. Proposed by the expert user community and subsequently approved by the central government, the biosafety level 2 (BSL-2) MX beamline is China’s first MX beamline equipped with P2 biosafety containment capabilities, fulfilling the country’s need for studying the crystal structures of infectious viruses and enhancing researchers’ capacity to conduct such investigations. The BSL-2 MX beamline at SSRF was established as part of the SSRF Phase II project (Xu *et al.*, 2023 ▸), which was initiated in December 2016. In compliance with the national standard ‘General Requirements for Laboratory Biosafety’ (GB19489-2008) and recommendations from research groups across China, a total of 39 specific virus species have been approved for experimental activities at this beamline. This beamline has been certified by the Shanghai Pudong Municipal Health Commission to perform BSL-2 MX experiments. X-rays are delivered into the beamline endstation, ensuring safety for both radiation and biosafety during the collection of diffraction data from virus samples in crystal form.

**Figure 6:**
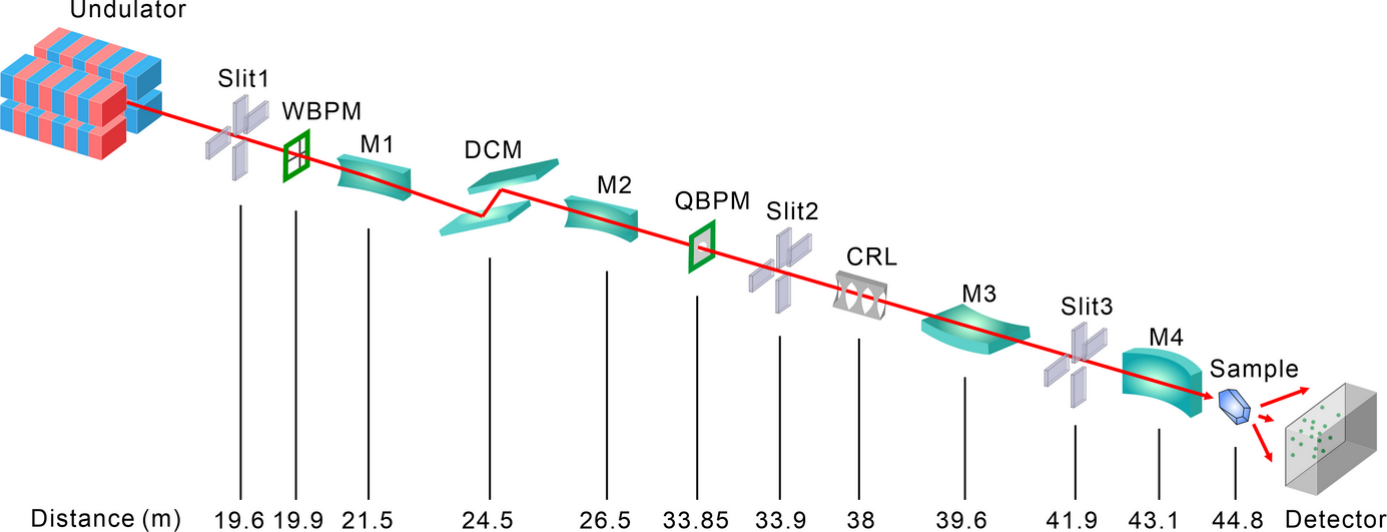
Schematic layout of the BL10U2-beamline. WBPM: wire beam position monitor; QBPM: quadrant beam position monitors; CRL: compound refractive lens.

**Figure 7:**
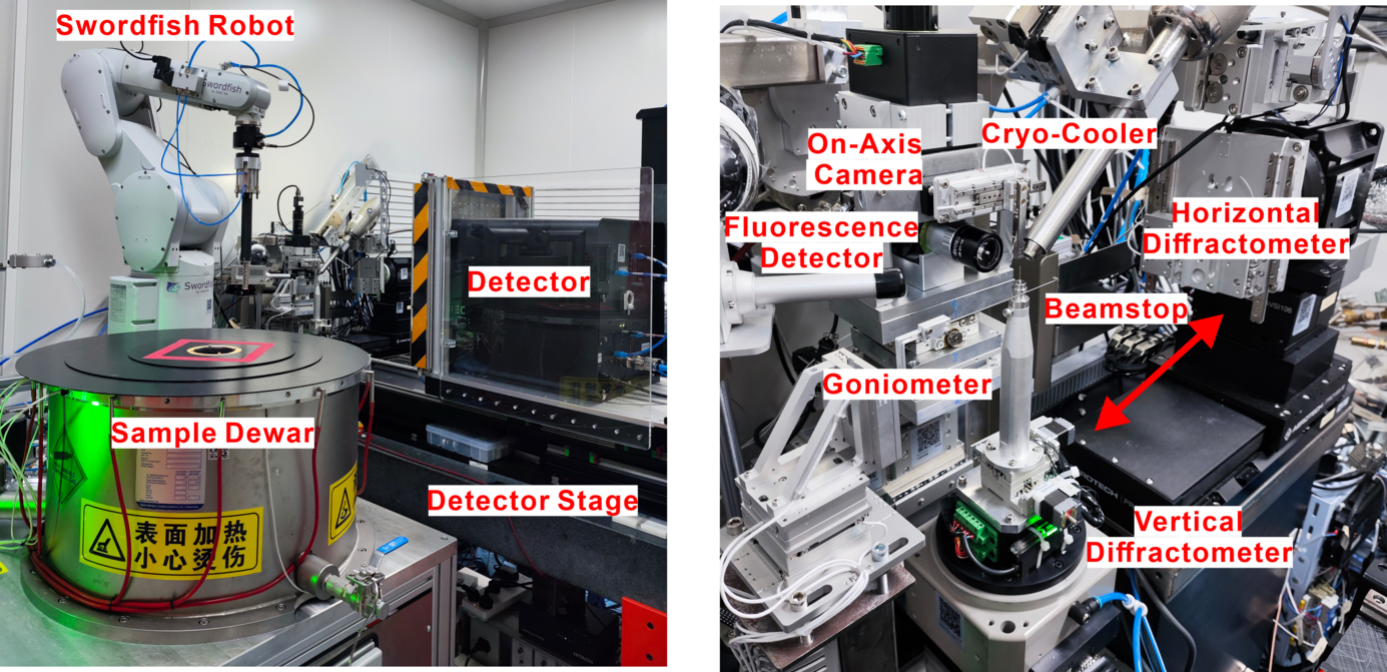
Endstation of the BL10U2 beamline.

BL10U2 accommodates a variety of experimental methods, including MX studies conducted under BSL-2 conditions, small crystal MX studies, serial crystallography techniques and anomalous diffraction methods (MAD/SAD), as well as IR and MR. To meet the requirements for samples from virus proteins and small crystal diffraction experiments – specifically regarding energy range, resolution, beam size, divergence angle and photon flux – the undulator IVU22 with 72 periods serves as the light source. The beamline’s optical design features a two-stage horizontal and single-stage vertical focusing system, utilizing precision slits and mirrors to optimize beam alignment and achieve focused beam dimensions at the sample point for enhanced experimental flexibility and BSL-2 protection within the radiation-shielding hutch (Fig. 6 ▸). Traditional cryo-crystallography experiments and *in situ* crystallography experiments are conducted using a dual-function interchangeable diffraction instrument. The dual-function interchangeable diffraction instrument operates through a transition between two sets of diffractometers. Two high-precision diffractometers, developed in-house (one horizontal for conventional cryo-crystal diffraction experiments and the other vertical for *in situ* crystal diffraction experiments) were installed on a stand separate from the detector support table. This configuration ensures minimal disruption to sample beam alignment during detector movement. Data collection is facilitated by a large-area, high-speed two-dimensional detector (Eiger X 16M), while sample mounting is streamlined through an automated changer (Swordfish) to enhance efficiency and stability. Data acquisition is managed via the in-house-developed *Finback* user interface. An overview of the endstation and the environment surrounding the sample is presented in Fig. 7 ▸. The parameters of the beamline are listed in Table 3 ▸.

Located within SSRF, the compact space of the beamline contrasts with its intricate experiment equipment. The endstation, spread across 35 m^2^, is divided into five rooms for sample transfer, personal protective equipment (PPE) donning, sample mounting and data collection. Constructed with lead-containing materials to minimize X-ray exposure, the endstation features nested hutch configurations. Operation guidelines require strict adherence to radioactive procedures, limiting user–sample contact post-mounting with equipment control facilitated through automated systems.

**Figure 8:**
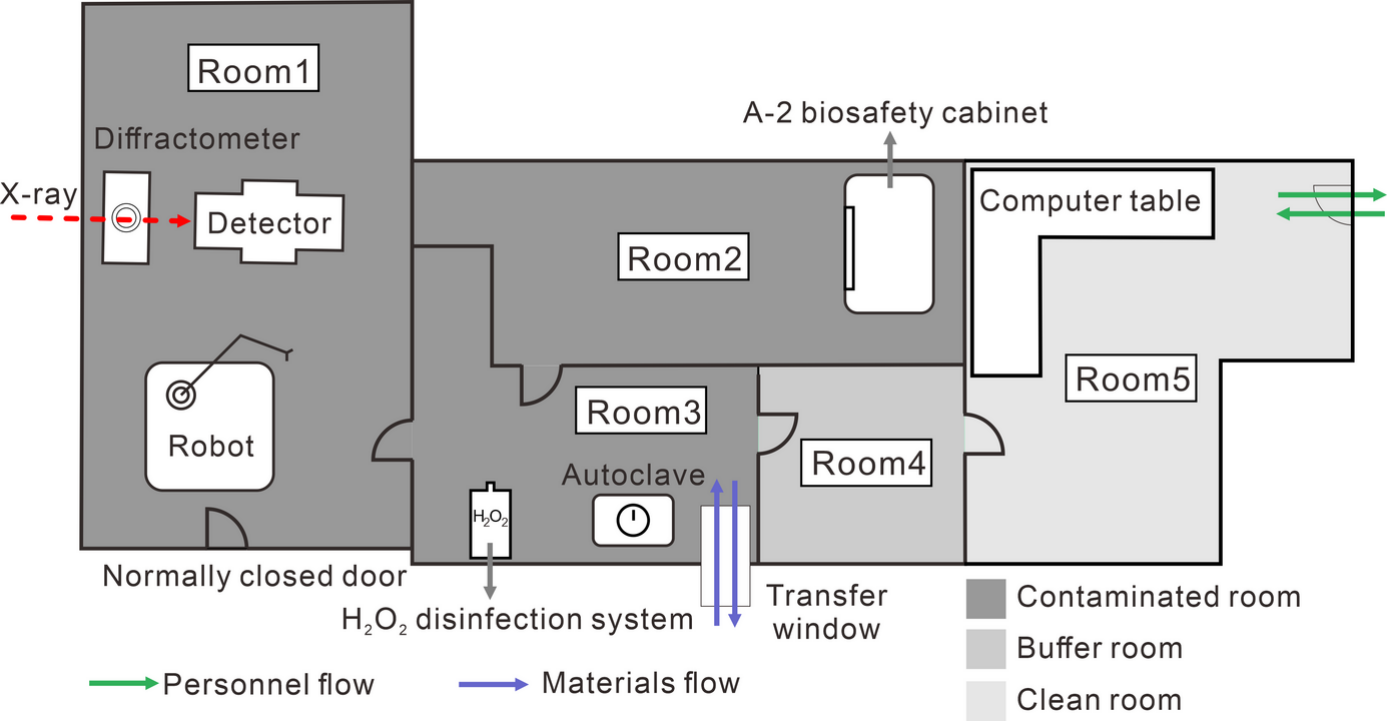
Layout of the endstation of BL10U2.

The five rooms of the endstation are tailored for specific tasks: room 1 serves as the experimental space; room 2 is equipped for plate inspection within a biological safety cabinet; room 3 is designated for sample transfer; room 4 is reserved for PPE change; and room 5 is utilized for data collection. Stringent environmental controls maintain temperatures in the range 19–25°C and humidity levels 45–65%, in compliance with national biosafety standards. These conditions are achieved through the use of negative pressure gradients and HEPA filters to ensure optimal air quality and safety. The experimental station features two doors: one door is exclusively designated for equipment transfer and remains closed during experiments, while the other door is equipped with access control measures to regulate operator entry. A transfer window is utilized to introduce samples into the laboratory and to transport biohaza­rdous samples out (Fig. 8 ▸).

Samples must be crystallized in a certified wet laboratory prior to experiments for structural determination. User proposals undergo rigorous evaluation by an interdisciplinary committee that assesses experimental details, pathogenic agents, associated risks, emergency protocols and personnel qualifications. Novel pathogens require additional scrutiny from the biosafety committee. Approved users must complete training, adhere to regulations and schedule experiments in accordance with the BSL-2 operating mode, following the guidance of the SSRF biosafety framework (Kong *et al.*, 2021 ▸). In BSL-2 mode, data on virus crystals are collected through X-ray irradiation using crystallization plates. Visual inspections are enhanced by on-axis microscopy and grid scans to improve data quality. Post-experiment sterilization procedures include autoclaving samples and decontaminating equipment and spaces using UV light or hydrogen peroxide vapor treatments.

The beamline commenced trial operations in June 2021, accumulating a total of approximately 7300 h of user-utilized time. By August 2024, 793 successful experiments involving 210 research groups from 108 institutions were completed, leading to impressive publications. Notable papers detailing the molecular structures of the Omicron and Delta variants of SARS-CoV-2 were published in high-impact journals such as *Cell* (Han *et al.*, 2022 ▸; Li *et al.*, 2022 ▸) and *Nature* (Duan *et al.*, 2023 ▸), providing valuable insights for vaccine and drug development against COVID-19. Additionally, industrial users were supported, receiving 609 h of beam time from the beamline. Through the support of this beamline, 146 structures have been resolved and deposited in the PDB to date.

### 2.4. BL17UM, a high-performance membrane protein crystallography beamline

In the rapidly advancing field of structural biology, certain proteins of significant importance pose challenges to researchers due to their unique structures and the difficulties associated with crystallizing them. A major obstacle in protein crystallography is the growth and screening of protein crystals, which has prompted a substantial investment of time and resources. The high-performance membrane protein beamline BL17UM is dedicated to determining the structures of proteins, such as membrane proteins, that produce only small crystals, in the 1 µm size range. The establishment of this beamline will significantly alleviate the challenges associated with the growth of larger crystals. Its design and construction were completed through a collaborative effort between SSRF and researchers from Shanghai Tech University.

**Figure 9:**
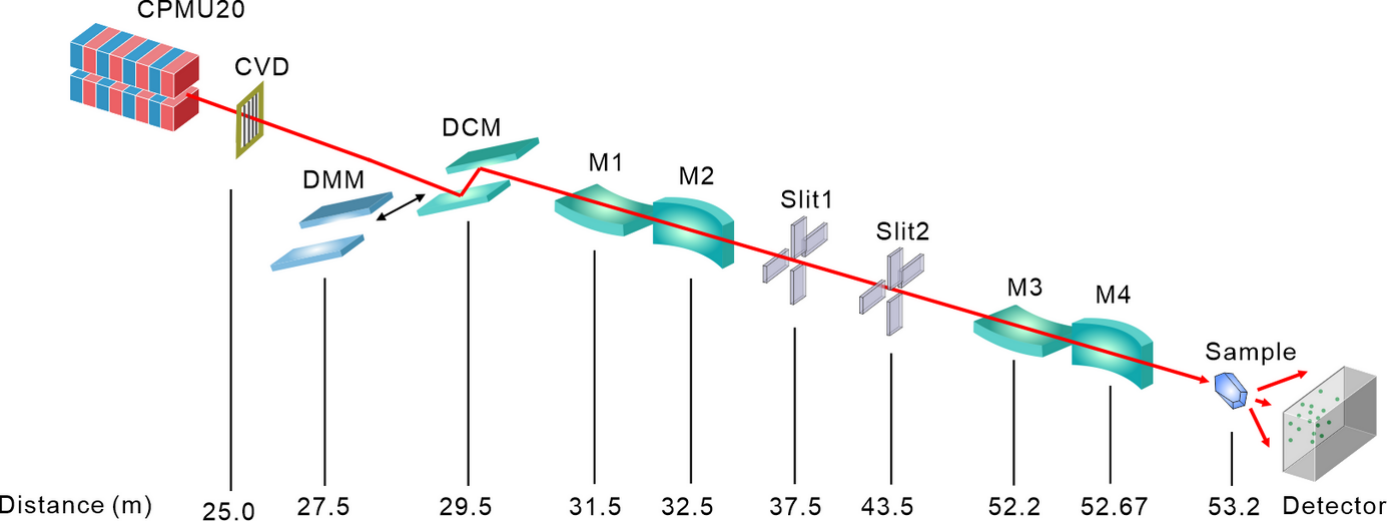
Schematic layout of the BL17UM beamline.

This beamline is the first in China to feature both a double multilayer monochromator (DMM) (Zhang *et al.*, 2024 ▸) and a DCM for interchangeable use (Fig. 9 ▸), expanding the energy range to 5–25 keV. Considering various aspects and experimental applications in the high-energy range, the DMM in BL17UM employs a Ru/C coating for the energy range of 10–18 keV and a W/Si coating for the 18–25 keV range. Each coating consists of 100 periods, with a thickness of 3 nm per period, resulting in an energy bandwidth of 0.775%, achieved through scanning with Si111 crystals. It achieves a photon flux exceeding 1.6 × 10^12^ photons s^−1^, with a sample spot size of less than 1 µm (horizontal and vertical dimensions of 0.7 µm), setting a record for photon flux density among similar Chinese beamlines and matching the advanced standards of international counterparts (Table 4 ▸).

**Figure 10:**
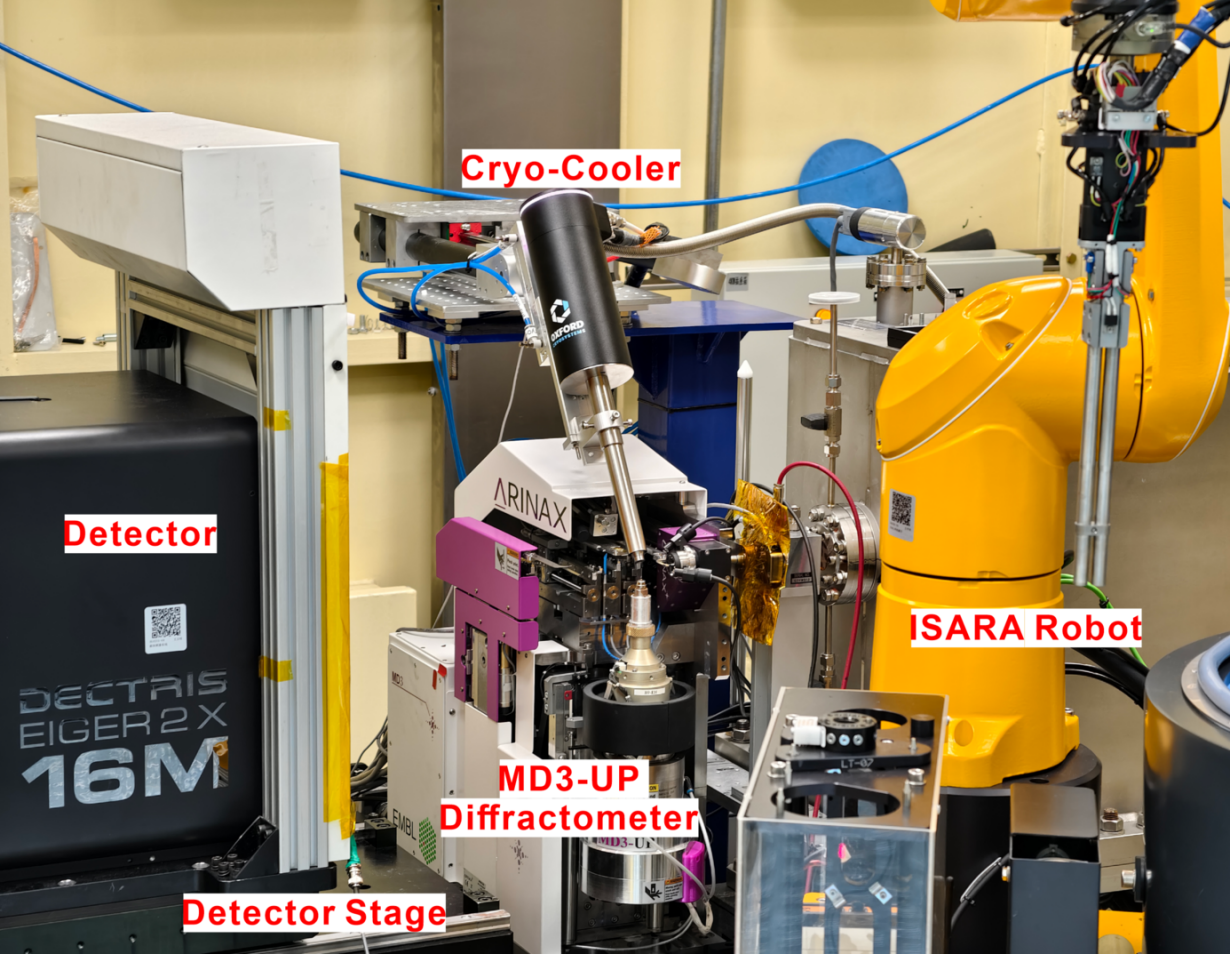
Endstation of the BL17UM beamline.

**Figure 11:**
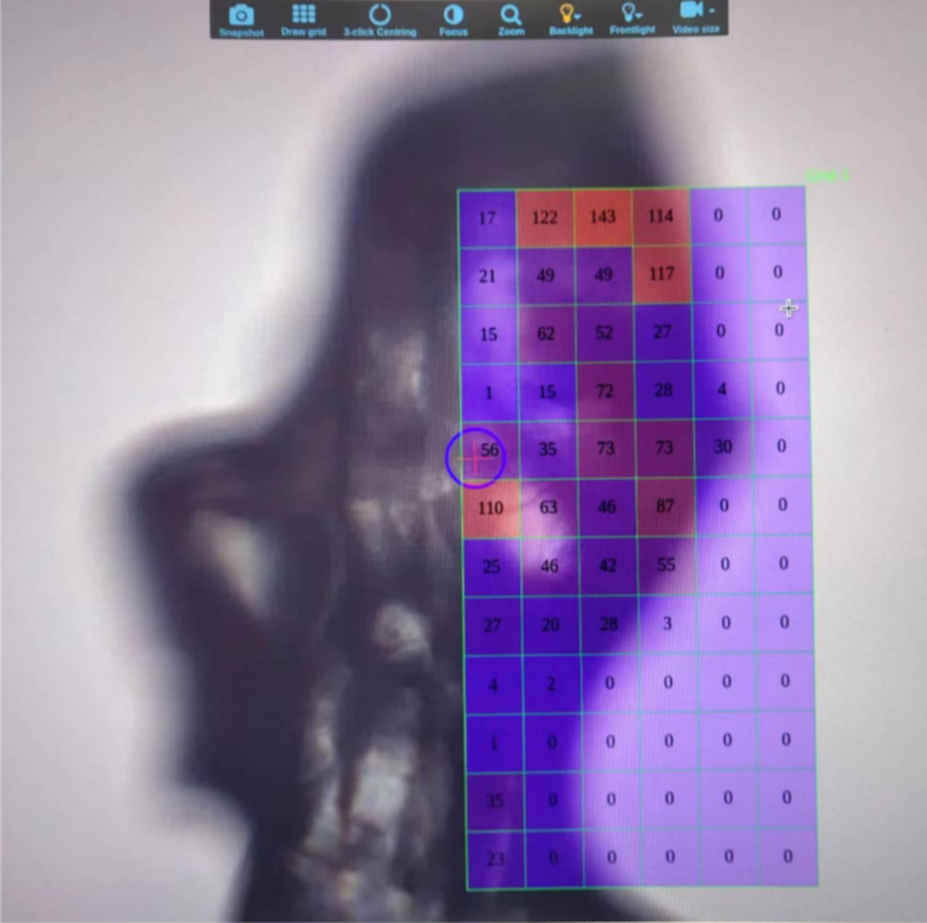
Raster data collection by *MxCube3*. A heat map is generated that represents the diffraction intensity.

Furthermore, BL17UM is equipped with advanced experimental instruments, including the Eiger2 X 16M area detector from Dectris, which features an active area of 311.2 × 327.8 mm and a maximum frame rate of 140 Hz; the MD3-UP diffractometer manufactured by ARINAX, with a sphere of confusion (SOC) of ≤0.1 µm, ensuring that small crystals remain within the X-ray focus spot; and the new dual-arm grasping robotic system produced by IRELEC, which enables a sample screening speed of over 49 crystals per hour (Fig. 10 ▸). The data acquisition system at the BL17UM experimental station utilizes the *MxCube3* system (Mueller *et al.*, 2017 ▸), a popular control and data acquisition platform for crystal diffraction beamlines based on a web interface. *MxCube3* allows for high-performance data acquisition through integrated control of the MD3-UP diffractometer, ISARA robot arm and Eiger2 X 16M detector. Its operational interface provides real-time status updates of the beamline and facilitates automatic sample loading (Fig. 11 ▸). Currently, the structural biology experimental methods supported by the BL17UM beamline include high-performance micro-focused beam protein crystallography, raster data collection, spiral stepping scan experiments and automated data processing.

The BL17UM beamline, equipped with high-performance devices, offers a robust platform for the structural analysis of micrometre-scale protein crystals at SSRF. It provides optimal experimental conditions for studying structurally challenging proteins, such as G-protein-coupled receptors (GPCRs), significantly reducing research cycles for protein crystal structures, conserving resources invested in crystallization and enhancing the efficiency of structural biology research. The beamline commenced trial operations in March 2022 and has since accumulated approximately 3200 h of user-utilized time. By October 2024, it facilitated 163 successful experiments involving 80 research groups, resulting in numerous impactful publications. Notably, this research includes the determination of X-ray crystal structures for both the ligand-free form and the NAD complex of the short-chain de­hydrogenase/reductase SsSDR1-WT (Che *et al.*, 2024 ▸), which identified critical sites influencing substrate selectivity. Furthermore, the rational redesign of the C1 and C2 cavities of SsSDR1 led to the development of the mutant I144A/S153L, significantly enhancing its activity towards α-halogenated aceto­phenones. In total, 16 structures have been resolved and deposited in the PDB, underscoring the beamline’s contributions to both academic and industrial research.

### 2.5. BL03HB, a Laue micro-diffraction beamline

**Figure 12:**
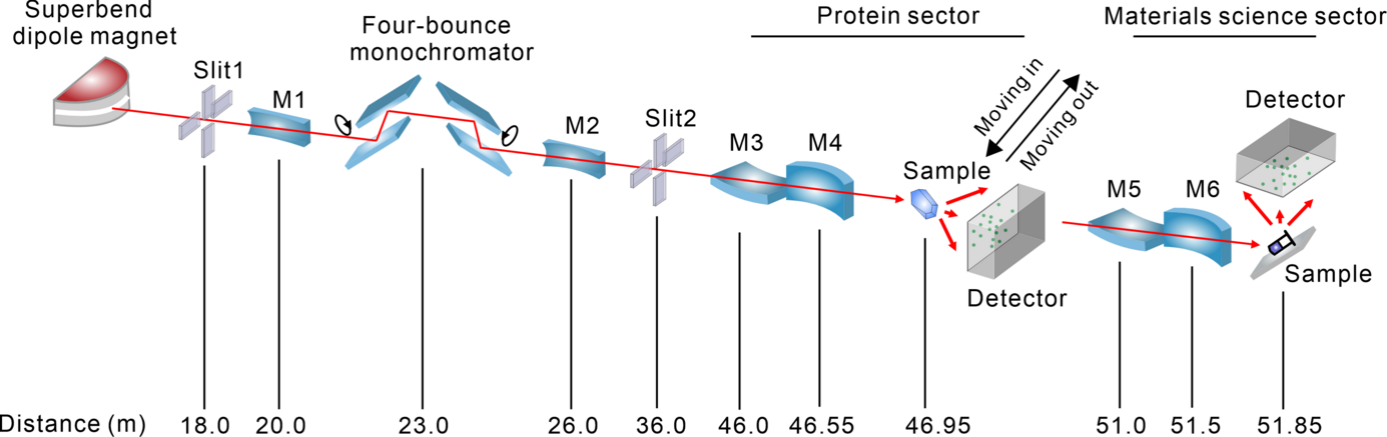
Schematic layout of the BL03HB beamline. When both channel-cut Si (111) crystals are aligned parallel to the optical path, the white beam passes directly through the openings of the two crystals. This configuration allows the monochromator to deliver both monochromatic X-rays and a white beam along the same path by simply rotating the two crystals.

Recent advancements in highly brilliant X-ray sources, fast-frame-rate detectors and innovative sample delivery techniques have generated significant interest in white-beam Laue microdiffraction. This method is effective for determining protein crystal structures using only a few diffraction images, allowing for room-temperature measurements and near-instantaneous data collection for time-resolved investigations of structural changes under physiological conditions. A Laue microdiffraction beamline (BL03HB) has been constructed at SSRF. Commissioned in June 2023, the BL03HB beamline utilizes a superbend dipole magnet with a magnetic field strength of 2.29 T and features a two-stage focusing system employing switchable K-B mirrors (Fig. 12 ▸). The beamline is divided into two sectors: one dedicated to MX, where white-beam Laue diffraction is collected using a Pilatus 2M area detector, and another focused on materials science, which collects reflection geometry data from sample surfaces with a second Pilatus 2M area detector (Wang *et al.*, 2024 ▸). The protein and materials science sectors are situated within the same experimental hutch, aligned in the direction of the X-ray beam, with the protein sector positioned first, followed by the materials science sector. When the materials science sector is in operation, all instruments, including the detector in the protein sector, can be moved out of the X-ray light path. A vacuum pipe is set up between the two K-B mirror chambers directly, ensuring vacuum conditions.

**Figure 13:**
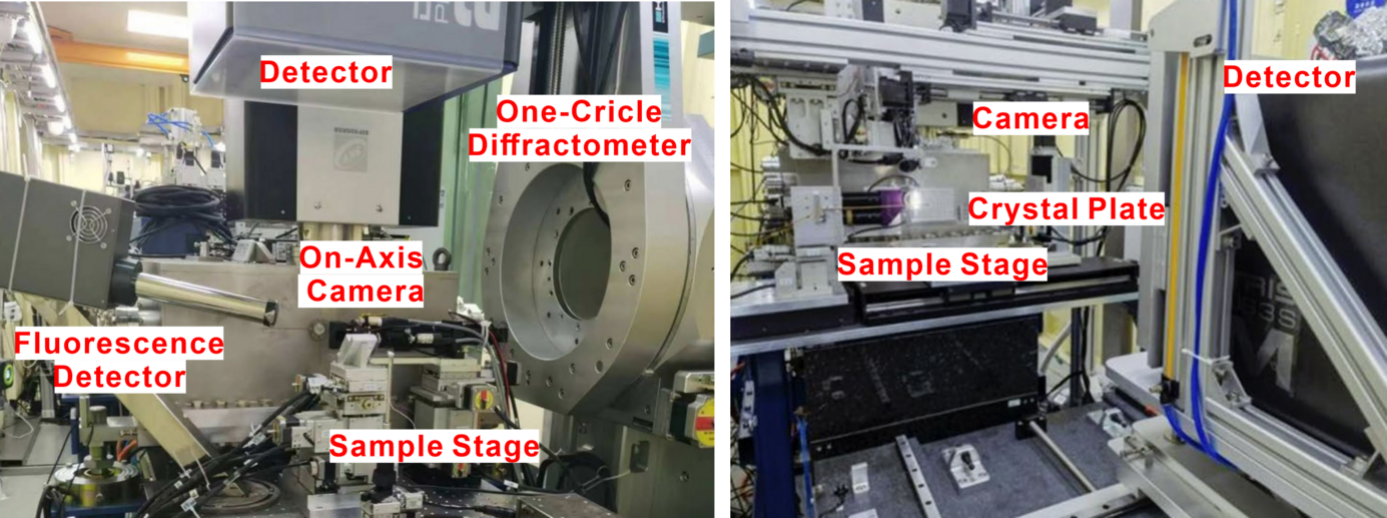
Left: diffraction experimental for nickel-based superalloys in the materials functional area. Right: pump–probe experiment for protein structure conducted in the protein functional area.

At standard operating conditions, the microbeams can achieve dimensions as small as 4.2 µm × 4.3 µm in the protein sector and 0.9 µm × 1.3 µm in the materials science sector (Table 5 ▸). The ability to easily switch between monochromatic and white beams enhances experimental versatility. The monochromatic beam is used for the determination of cell parameters at room temperature, because the cell parameters at room temperature are different from those at cryo-temperature; since it is difficult to obtain the cell parameters with the white beam, the monochromatic beam is used for measuring the cell parameters at BL03HB. The control system for the Laue protein crystal *in situ* diffraction experiment employs the *BluIce/DCS* data acquisition system. Additionally, a locally developed MATLAB-based program, known as *Laueprocess*, is utilized for analyzing the collected Laue diffraction patterns from protein crystals, based on the *Lauegen* software (Campbell, 1995 ▸; Campbell *et al.*, 1998 ▸; Hao *et al.*, 2021 ▸). Currently, BL03HB supports techniques primarily focused on high-temperature nickel superalloys and pump–probe Laue crystallography (Fig. 13 ▸). In summary, BL03HB significantly advances the application of Laue microdiffraction in both MX and materials science.

The beamline commenced trial operations in June 2023, accumulating a total of approximately 3900 h of user-utilized time. By August 2024, the beamline had completed 85 user projects, with experiments in fields such as high-temperature nickel superalloys and pump–probe Laue crystallography, which supported 40 Science Citation Index (SCI) papers. More than 20 from 85 experiments were performed for Laue crystallography experiments. We are planning to improve BL03HB by implementing a microfluidics reaction system.

### 2.6. User community and output

The MX beamlines mentioned above serve a stable and growing user community, currently comprising over 300 independent research teams. A significant portion of these users is from the Yangtze River Delta region, with approximately 120 teams utilizing the MX facilities. Other notable contributors to the user community include about 60 teams from the Beijing–Tianjin–Hebei region, around 50 from central and western regions, and approximately 40 from the Pearl River Delta region. Users can conduct their experiments on-site or remotely (Wang, Sun *et al.*, 2019 ▸). SSRF not only supports domestic user experiments but also actively seeks to attract international users. International users can apply for beam time through the website at https://ssrfwx.ssrf.ac.cn/proposals/en/a/login.

Presently, approximately 10% of beam time is allocated to corporate users. To meet the high demand from this diverse user base, customized service schemes are available. In addition to traditional MX research, there is also a small but growing group of researchers engaged in projects involving small organic and inorganic molecules utilizing the MX beamlines.

The scientific achievements of the MX beamlines are clearly demonstrated by the statistics on depositions in the Protein Data Bank (PDB). The first structure derived from data collected at SSRF was published in 2009 (PDB code 3id4, Li *et al.*, 2009 ▸). As of October 2024, scientists have resolved over 5000 protein structures using the SSRF MX beamlines (Table 6 ▸).

The most recent reliable data on publications span from 2009 to 2023, during which over 2500 scientific papers were published, including 108 in prestigious journals such as *Nature*, *Science* and *Cell*. Notably, in 2015, the world’s first crystallographic analysis of the human glucose transporter 1 (GLUT1) was completed, providing critical insights into the transport mechanisms that facilitate the movement of essential substances across cellular membranes, as well as elucidating its operational mechanisms and the pathological processes related to associated diseases (Deng *et al.*, 2014 ▸). In 2019, researchers determined the high-resolution structure of the primary light-harvesting complex protein (FCP) from diatoms at a resolution of 1.8 Å, yielding the first definitive experimental evidence regarding the aggregation states of major light-harvesting proteins, an issue that had been debated for decades. This study also offered innovative insights and strategies for designing new crop varieties aimed at enhancing light absorption and photoprotection efficiency in plants (Wang, Yu *et al.*, 2019 ▸). Additionally, other significant international contributions include investigations into the structural mechanisms of transcriptional activation-like effectors and their specific interactions with DNA (Deng *et al.*, 2012 ▸), as well as the identification of new drug targets and elucidation of mechanisms of action for anti-tuberculosis therapies (Zhang *et al.*, 2020 ▸). These accomplishments highlight the essential role of the SSRF MX beamlines in advancing research within local, national and international communities.

The opening of MX beamlines at SSRF has stimulated the development of innovative enterprises which focused on structure-based drug discovery. Several globally renowned pharmaceutical company R&D centers in China have used MX beamlines at SSRF to conduct research projects. Additionally, domestic innovative pharmaceutical companies and contract research organizations (CROs) in China have achieved significant successes in drug discovery, exemplified by the innovative drug Zanubrutinib, which was approved by the US FDA (Guo *et al.*, 2019 ▸).

## 3. Technological developments

### 3.1. MX data collection system

**Figure 14:**
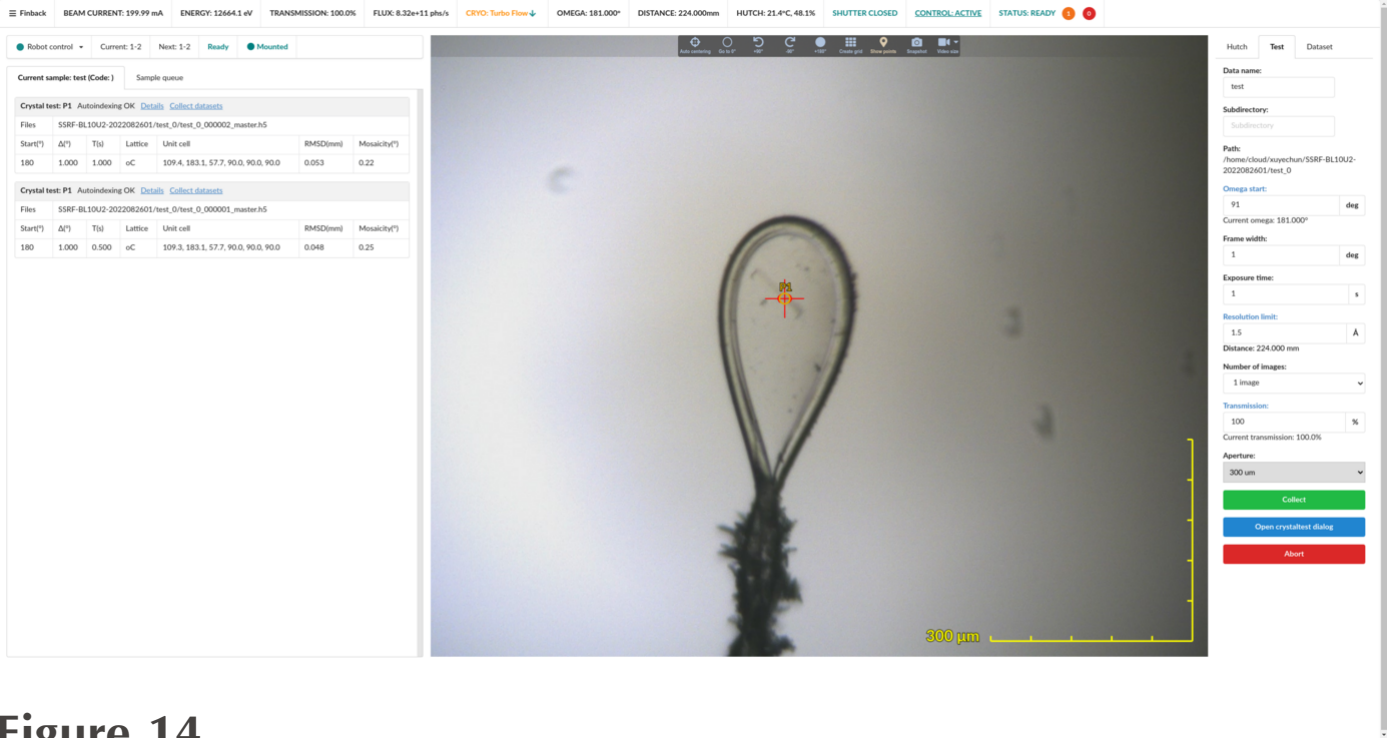
The *Finback* GUI.

Inspired by the newly developed MX data collection system in recent years, particularly *MXCube3* (Mueller *et al.*, 2017 ▸), we designed *Finback*, a new MX data acquisition system with a user-friendly web-based graphical user interface (GUI) for interactive data collection (Fig. 14 ▸). Since June 2021, *Finback* has been implemented at SSRF BL02U1 and BL10U2, facilitating simultaneous access for both on-site and remote users.

The *Finback* GUI operates on modern web browsers and has been developed using contemporary web technologies, including *WebSocket*, *WebGL*, *Web Worker* and *WebAssembly*. It supports multiple concurrent sessions and integrates seamlessly with the experimental data management system, automatically depositing relevant experimental parameters and results into a database. To accommodate various beamlines, *Finback* configures numerous parameters, such as beam center, exposure time limits and sample changer positions. These parameters are typically stored in files or databases but require a program restart for modifications to take effect. *Finback* addresses this by directly utilizing *EPICS* for parameter settings, eliminating compatibility concerns beyond *EPICS* drivers. With approximately 200 configuration parameters (excluding hardware), each corresponding to a process variable (PV), irregularly changing parameters like beam center and sample positions benefit from *EPICS*’ autosave module for regular backup and restoration. Using *EPICS*’ callback mechanism allows real-time updates of parameter changes across *Finback*’s frontend and backend, preventing the need for restarts before the updated parameters take effect.

### 3.2. Automatic data processing pipeline and experiment information management system

**Figure 15:**
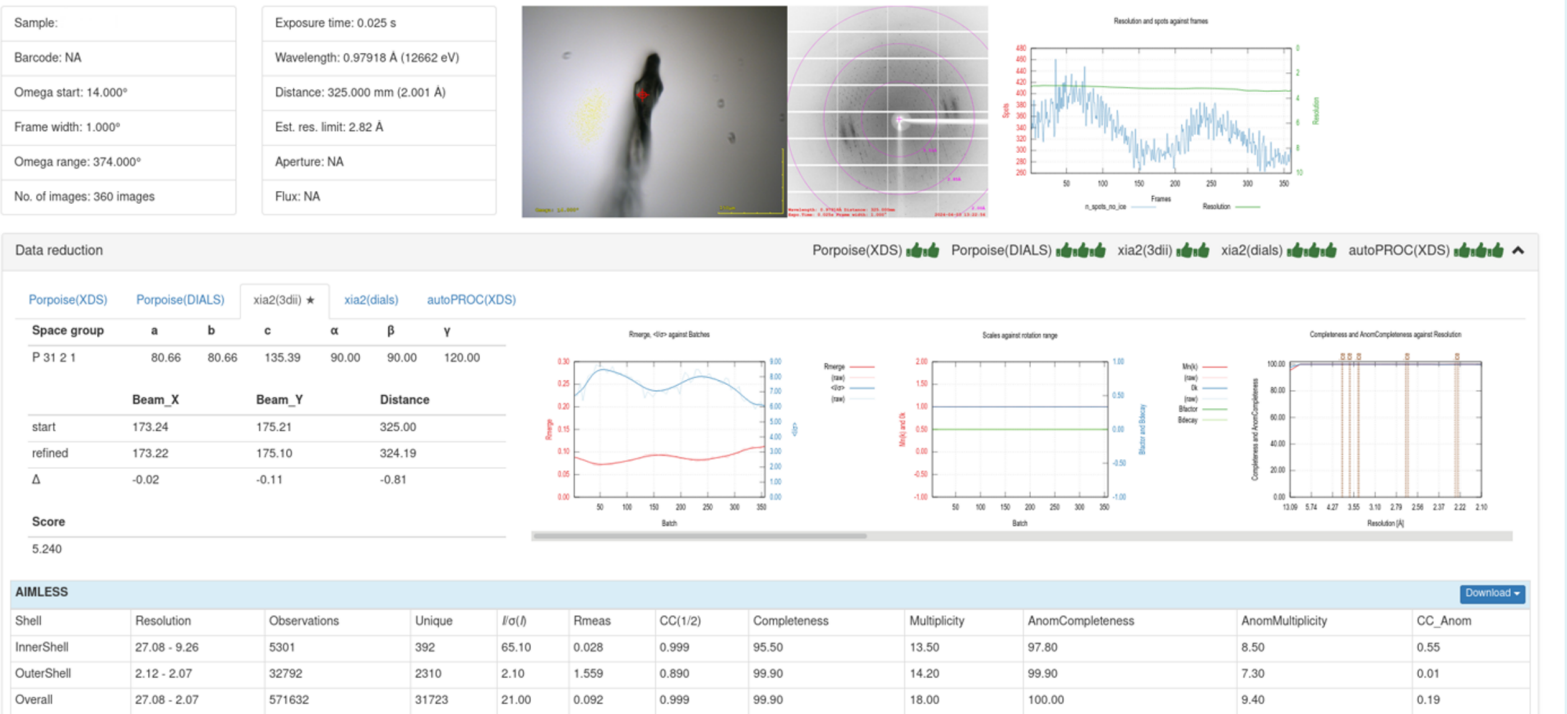
The data reduction result view of SealWeb.

With the advent of hybrid pixel array detectors such as Pilatus and Eiger, the volume of diffraction data collected has significantly increased, particularly with the implementation of data collection techniques like fine-slicing, which enhances the signal-to-noise ratio. Manual processing of these extensive datasets is inefficient and susceptible to errors, complicating the assessment of data quality when hundreds of datasets are generated daily. To tackle this issue, we developed and deployed an automated data processing and experiment information system for the SSRF MX beamline, which encompasses data reduction, SAD phasing and a web platform known as SealWeb. By leveraging the high-performance computing cluster at SSRF, user data processing can be completed rapidly, and users can monitor the data processing results and progress through the website (Fig. 15 ▸). Furthermore, both the data collection system and the data processing system are integrated with the SSRF user account system, ensuring that users can only access data associated with their own accounts.

### 3.3. FBDD development

**Figure 16:**
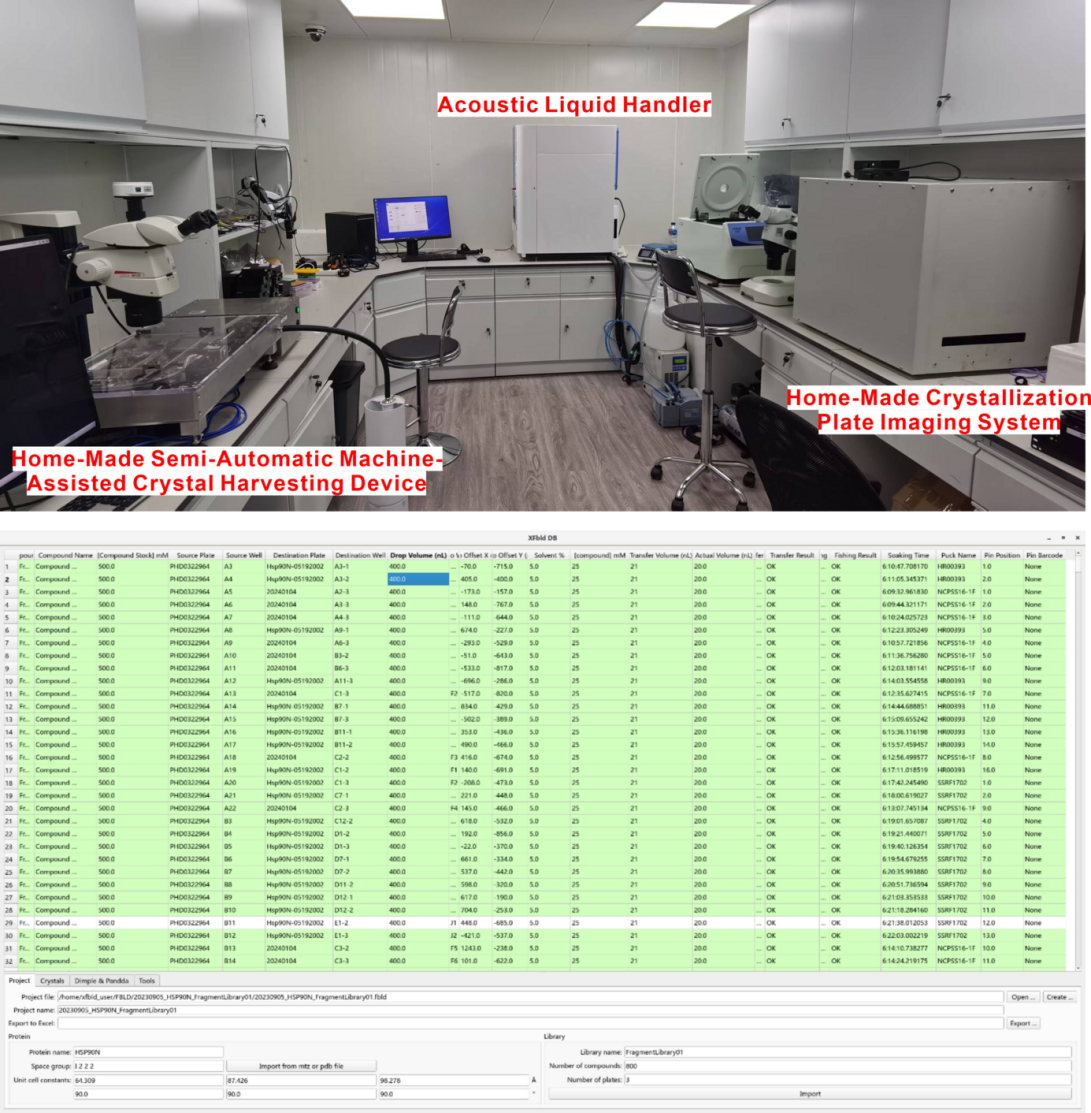
Top: the high-throughput complex crystal sample preparation system for FBDD platform. Bottom: the experimental information management software for FBDD screening.

The fragment-based drug discovery (FBDD) platform utilizes X-ray crystallography for comprehensive fragment screening on a global scale. This technology significantly accelerates the drug discovery process. The platform at Diamond Light Source (DLS) serves as a successful example of aiding COVID-19 drug discovery (Walsh *et al.*, 2021 ▸; Boby *et al.*, 2023 ▸). A high-throughput FBDD platform based on protein crystallography has been established by integrating a complex crystal sample preparation system – comprising a non-contact acoustic liquid handler, a home-made automatic crystallization plate imaging system and a semi-automatic machine-assisted crystal harvesting device – with beamlines, experimental information management software and a high-throughput structure analysis pipeline. This platform enables the rapid preparation of protein fragment compound complex crystals, data collection and structural analysis (Fig. 16 ▸). Currently, we offer two types of compound libraries: the Enamine DSI-Poised Library, which contains 860 compounds, and our custom library, comprising 800 compounds. We have developed a comprehensive suite of experimental technologies that encompasses high-throughput preparation of crystalline complexes and the identification of fragment molecules. With this array of experimental solutions, the platform achieves rapid and efficient fragment screening, thereby advancing structure-based drug development. In the future, we will focus on employing ‘body temperature (37°C) MX’ to investigate the potential binding ligands (Jacobs *et al.*, 2024 ▸). Additionally, we will assess the kinetic stability of the hydration network within the structure to achieve more reliable and insightful results.

### 3.4. SSX development

Serial synchrotron crystallography (SSX) is a rapidly evolving field (Nam, 2024 ▸) which adapts the concept of serial crystallography from free-electron laser (FEL) facilities (Hough & Owen, 2021 ▸). Currently, nearly all micro-focus MX beamlines worldwide can facilitate some form of SSX experimentation (Pearson & Mehrabi, 2020 ▸). At SSRF, several sample delivery instruments have been developed to support the SSX method.

For fixed-target delivery, an *in situ* microplate (Liang *et al.*, 2020 ▸) has been designed to integrate crystal growth and data collection. This microplate eliminates the need for crystal harvesting, as it can be secured to a sample base and mounted on the goniometer head, thus allowing compatibility with standard cryo-crystallography operations. The fast grid scan program facilitates efficient SSX data collection with this fixed target. To further enhance efficiency, a novel combined crystallization plate (Liang *et al.*, 2021 ▸) was developed based on this microplate, enabling an automated experimental process from crystal growth to data collection.

Microfluidics also serves as a promising technique for sample delivery, with recent results reported (Monteiro *et al.*, 2020 ▸). SSRF has introduced a microfluidic rotary-target sample device (Zhao *et al.*, 2020 ▸) that allows adjustable rotation speeds based on experimental parameters and can be easily mounted on the goniometer head. This device comprises a microfluidic sample plate and a motion control system. The microfluidic sample plate features a seven-layer sandwich structure that facilitates *in situ* growth and diffraction of protein crystals, while also offering low background scattering and minimal X-ray absorption. A motor integrated with a goniometer is employed as the motion control system. This device offers several significant advantages, including a wide adjustable range of delivery speeds, low background noise and minimal sample consumption.

### 3.5. Hardware development

**Figure 17:**
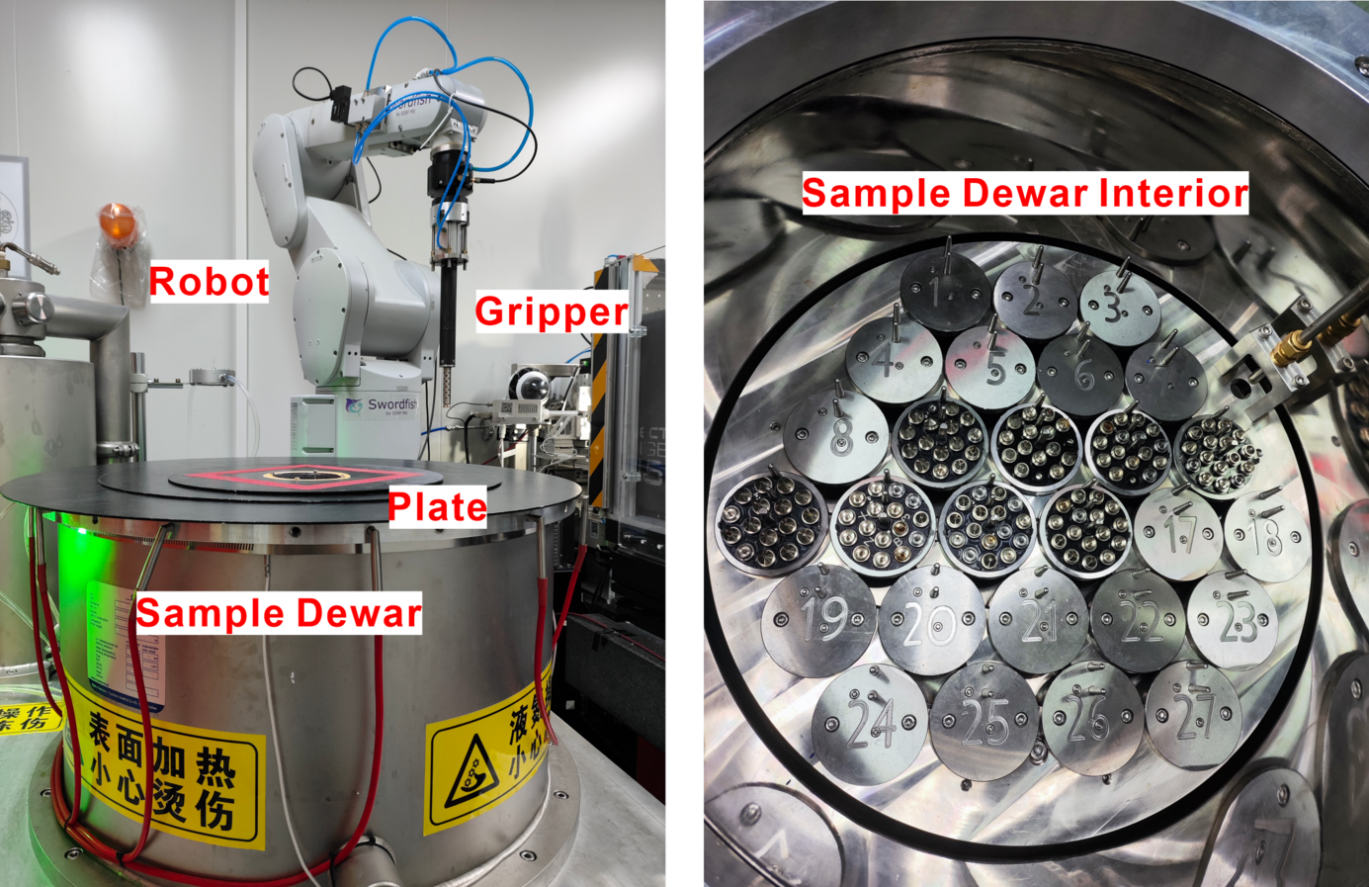
The Swordfish automated sample changer. Left: overview. Right: the interior of the sample Dewar.

Recent advancements in high-speed X-ray detectors and beamline automation have highlighted the critical need for efficient sample management in MX experiments. To address this demand, we have developed an automated sample changer, named Swordfish, which incorporates essential features such as rapid operation, substantial sample storage capacity, reliability and user-friendliness. The Swordfish sample changer crystal exchange manipulator consists of three components: a large-capacity sample Dewar, a high-stability sample gripper and user-friendly control software. A three-point positioning method for the puck was employed, significantly simplifying the placement and removal of pucks in a liquid-nitro­gen environment. The bottom drivers and input/output controller (IOC) for the commercial robotic arm were developed within the *EPICS* environment. Additionally, a user-friendly web-based GUI was designed as part of the *Finback* software. This automated sample changer is specifically designed for MX applications and can efficiently transfer a crystal sample from the goniometer to a storage Dewar that accommodates up to 432 samples (27 pucks) in approximately 20 s. A barcode scanner is utilized to capture crystal information, thereby enhancing record-keeping during high-throughput screening. For optimal performance, a standard pin measuring 18 mm in length is recommended (Fig. 17 ▸).

A set of high-precision diffractometers, one horizontal for conventional cryo-crystal diffraction and one vertical for *in situ* crystal diffraction, have been developed in-house (Sealion). Sealion features air-bearing goniometers with a SOC of less than 1 µm, a high-precision sample centering system, an on-axis microscope, a collimator, backlight, an ultrafast shutter, fluorescence detectors and interchangeable collimators. The on-axis microscope achieves a resolution of up to 900 line-pairs per millimetre (lp mm^−1^) within a field of view (FOV) measuring 0.14 × 0.19 mm at maximum magnification, and a resolution of 270 lp mm^−1^ within an FOV of 1.7 mm × 2.3 mm at minimum magnification. A working distance of 34 mm allows for adequate space to securely mount samples. The collimator is constructed from two tantalum blocks featuring holes of varying sizes (50 µm and 300 µm) designed to minimize stray beams. Furthermore, a moveable beamstop (Pan *et al.*, 2014 ▸) has been strategically placed after the sample to block any remaining radiation.

## 4. Peripheral laboratories

The Biology and Biomedical Laboratory plays a vital role in supporting the capabilities of the above MX beamlines at SSRF, with key functions including protein crystal growth and storage, heavy-atom soaking and other related procedures that advance research efforts. The laboratory houses over 50 instruments associated with MX beamlines, including an isothermal titration calorimeter, spectrophotometer, circular dichroism analyzer, dynamic light scattering analyzer, PCR equipment, high-pressure cell cracker, gel chromatograph, fast protein liquid chromatography system, automated crystal observation system, biochemical incubator, high-speed freeze centrifuge, high-pressure freezer, multifunction microplate reader, frozen tissue microtome, stereomicroscope, fluorescence stereomicroscope and imaging systems. Additionally, it features three dedicated cell culture rooms, a 4°C constant-temperature room and an 18°C constant-temperature room. Overall, the Biology and Biomedical Laboratory equips researchers to conduct comprehensive MX research at SSRF, establishing it as a hub for cutting-edge biological and biomedical investigations.

## 5. Conclusion and perspectives

The construction and technological advancements of the MX beamlines at SSRF, along with its consistent operation, have significantly propelled progress and innovation across related scientific research fields in China. Since the beamline became operational, the research team has rapidly expanded, forming a stable group of researchers who are actively investigating their respective domains. Their efforts have led to numerous influential research outcomes that have made substantial contributions to both academia and industry in China, and internationally via our publications and PDB depositions.

Looking ahead, a strategic emphasis will be placed on advancing serial crystallography and structure-based drug design. These areas not only provide fresh perspectives for fundamental scientific inquiry but also present extensive opportunities for the design and development of clinical drugs. By further optimizing experimental techniques and equipment, as well as enhancing data analysis capabilities, significant breakthroughs are anticipated in these fields, ultimately contributing to advancements in health and life sciences.

## Figures and Tables

**Table 1:** The dedicated MX beamlines in operation at SSRF

Source position	Beamline name	Status
BL17U1	Macromolecular X-ray crystallography beamline	Operation 04/2009–02/2021, decommissioned
BL02U1	Macromolecular X-ray crystallography beamline	Operation since 06/2021
BL10U2	BLS-2 macromolecular crystallography beamline	Operation since 06/2021
BL17UM	High-performance membrane protein crystallography beamline	Operation since 03/2022
BL03HB	Laue micro-diffraction beamline	Operation since 06/2023

**Table 2:** Specifications of the BL02U1 beamline

Equipment	Specifications
Light source	Undulator, 22 mm × 69 periods, 6–12 mm gap
Monochromator	Cryo-cooled double flat crystal Si111
X-ray energy range (keV)	6–16
*K*-value range of undulator (6–16 keV)	1.588–1.810
Wavelength range (Å)	0.77–2.07
Energy resolution (Δ*E*/*E*, @12.7 keV)	<2 × 10^−4^
Flux at sample (@12.7 keV, 300 mA) (photons s^−1^)	4.5 × 10^12^
Focused beam size (FWHM) (µm)	11.6 × 4.8 (H × V)
Typical beam divergence (mrad)	1.3 × 0.25
Goniometer	Single horizontal rotation axis diffractometer
Cryo capability (K)	100
Sample mounting	Swordfish automated sample changer/manual
Detector model	Eiger2 S 9M

**Table 3:** Specifications of the BL10U2 beamline

Equipment	Specifications
Light source	Undulator, 25 mm × 72 periods, 6–12 mm gap
Monochromator	Cryo-cooled double flat crystal Si111
X-ray energy range (keV)	7–18
*K*-value range of undulator (7–18 keV)	1.452–1.711
Wavelength range (Å)	0.69–1.77
Energy resolution (Δ*E*/*E*, @12.7 keV)	2 × 10^−4^
Flux at sample (@12.7 keV, 300 mA) (photons s^−1^)	2 × 10^12^
Focused beam size (FWHM) (µm)	20 × 10 (H × V)
Typical beam divergence (mrad)	1.5 × 0.2
Sample screening speed	>15 samples per hour
BSL-2 biosafety protection	Yes
Highest diffraction resolution	≤0.8 Å
Diffractometer	Dual-function interchangeable diffractometer
Cryo capability (K)	100
Sample mounting	Swordfish automated sample changer/manual
Detector model	Eiger X 16M

**Table 4:** Specifications of the BL17UM beamline

Equipment	Specifications
Light source	Undulator, 20 mm × 160 periods, 6–20 mm gap
Monochromator	Cryo-cooled double flat crystal Si111 (DCM)
	DMM
X-ray energy range (keV)	5–25 (DMM)
	10–25 (DCM)
*K*-value range of undulator (5–25 keV)	0.573–1.767
Wavelength range (Å)	0.49–2.48
Energy resolution (Δ*E*/*E*, DCM@12 keV)	2 × 10^−4^
Flux at sample	1.6 × 10^11^ photons s^−1^ (DCM@12 keV, 300 mA)
	1.6 × 10^12^ photons s^−1^ (DMM@12 keV, 300 mA)
Focused beam size (FWHM) (H × V, µm)	0.75 × 0.65 (DCM)
	0.70 × 0.68 (DMM)
Typical beam divergence (mrad)	2.5 × 1.0
Sample screening speed	>40 samples per hour
Highest diffraction resolution	≤0.6 Å
Goniometer	MD3-UP, single-axis
Cryo capability (K)	100
Sample mounting	ISARA automated sample changer/manual
Detector model	Eiger X 16M

**Table 5:** Specifications of the BL03HB beamline

Equipment	Specifications
Light source	A superbend dipole magnet with a magnetic field of 2.29 T
Monochromator	Four-bounce monochromator consisting of two channel-cut Si(111) crystals
X-ray energy range (keV)	6.538–20 (monochromatic)
	7–20 (white beam)
Energy resolution (Δ*E*/*E* @10 keV)	0.96 × 10^−4^ (monochromatic)
Flux at sample	1.95 × 10^10^ photons s^−1^ (DCM @10 keV, 300 mA)
	6 × 10^14^ photons s^−1^ (white beam, 300 mA)
Focused beam size (FWHM) (µm)	4.0 × 4.2 (H × V, DCM @10 keV, 300 mA)
	4.2 × 4.3 (H × V, white beam, 300 mA)
Typical beam divergence (mrad)	2.5 × 1.0
Highest diffraction resolution	1.3 Å
Goniometer	4D translation
Cryo capability (K)	100
Sample mounting	Manual
Detector model	Pilatus 2 M

**Table 6:** Number of PDB structures for which data were collected at SSRF MX beamlines

Year	BL02U1	BL03HB	BL10U2	BL17U1	BL17UM
2009	0	0	0	8	0
2010	0	0	0	113	0
2011	0	0	0	202	0
2012	0	0	0	334	0
2013	0	0	0	378	0
2014	0	0	0	334	0
2015	0	0	0	442	0
2016	0	0	0	395	0
2017	0	0	0	415	0
2018	0	0	0	363	0
2019	0	0	0	371	0
2020	0	0	0	371	0
2021	27	0	2	368	0
2022	195	0	50	240	0
2023	176	1	74	116	2
2024	36	2	20	24	14
Total	434	3	146	4474	16
